# Nanotribological Properties of Ga- and N-Faced Bulk Gallium Nitride Surfaces Determined by Nanoscratch Experiments

**DOI:** 10.3390/ma12172653

**Published:** 2019-08-21

**Authors:** Jian Guo, Changjun Qiu, Huiling Zhu, Yongqiang Wang

**Affiliations:** School of Mechanical Engineering, University of South China, Hengyang 421001, China

**Keywords:** gallium nitride (GaN), nanotribological properties, wear resistance, frictional coefficient, nanoscratch

## Abstract

Through nanoscratch experiments with a spherical diamond indenter, a contrastive study of the nanotribological properties of Ga- and N-faced gallium nitride (GaN) samples was carried out. Nanoindentation results revealed that the elastic modulus of the Ga-faced GaN sample was slightly higher than that of N-faced GaN sample. Particularly, Ga- and N-faced GaN samples exhibited rather different nanotribological properties, and the Ga-faced sample showed a stronger wear resistance. The study indicated that the critical normal load required to cause material removal of N-faced GaN sample was almost two times that of Ga-faced GaN sample. Both Ga- and N-faces exhibited a rather low frictional coefficient at the elastic and elastoplastic stages of material, e.g., ~0.06 for Ga-face and ~0.075 for N-face when scratching under the progressive normal load. Combined with transmission electron microscopy and X-ray photoelectron spectroscopy, we speculated that, except for the intrinsic atomic arrangements attributed to the non-reverse crystallographic symmetry of c-plane wurtzite GaN, the difference of nanotribological properties between Ga- and N-faces may also be related to the preferential formation of a native oxide layer and a slight lattice damage layer on the N-faced GaN surface. This study can enrich the understanding of the nanotribological properties of Ga- and N-polar-faced bulk monocrystalline GaN materials fabricated by the conventional technique.

## 1. Introduction

By virtue of its wide forbidden band, direct energy gap, high temperature, and pressure resistance, etc., gallium nitride (GaN) has been widely used in blue/green/ultraviolet light emitting diodes (LEDs) [1,2], high electron mobility transistors [3,4], high power and frequency electronic devices [5,6], and so on. During the processes of preparation, packaging, and transportation of GaN-based devices, e.g., LEDs, a dislocation or defect can be easily produced under the slight stress and creep effect (usually caused by the normal load and frictional force at micro/nanoscale) [7,8], which would lead to the reduction of photoelectric conversion efficiency of LEDs [9]. To reduce, or even avoid, the negative influences of friction and wear on the luminous performance of LEDs, the understanding of the nanotribological properties of GaN material is quite essential. In addition, mechanical damages in GaN epilayers, such as film cracking and interface delamination produced during the chemico-mechanical polishing (CMP) process, usually degrade the processing yield and the reliability of their applications in microelectronic devices [10]. Therein, the nanotribological properties (e.g., wear at nanoscale) of GaN material is a key factor influencing the material removal efficiency and lattice damage behavior. The study of nanotribological properties could help to understand the atomic-level CMP mechanism [11,12].

Previous relevant studies of GaN materials mostly focused on their mechanical properties [13,14] and CMP technology [11,12,15,16], but directly studying on their tribological properties was rarely reported. The existing experimental results on tribology were generally investigated on (0001) crystallographic plane GaN coating or films that were grown epitaxially on a monocrystalline sapphire substrate using the metalorganic chemical vapor deposition (MOCVD) method. Zeng et al. researched the tribological performance of GaN(0001) coating and found a remarkable physical property of GaN: When rubbed by a ruby microsphere with radius of ~750 μm, GaN exhibited an extremely low wear rate (10^−9^ to 10^−7^ mm^3^/(N·m)), approaching the lowest wear rates reported for diamonds ((10^−9^ to 10^−10^ mm^3^/(N·m) [17]. The authors also indicated that the tribochemical wear behavior of GaN was strongly dependent on the crystal orientation [18] and moisture [19]. Lin et al. studied the effect of crystal orientation on the nanoscratch characteristics of the GaN film’s surface, suggesting that the c-axis GaN epilayers had higher shear resistance than a-axis GaN epilayers [20]. The above results together indicated that no matter what rubbed against the high-hardness diamond or ruby, the GaN coating or film showed excellent wear resistance.

It is well known that the stable crystalline structure of GaN material is generally the hexagonal wurtzite, inside which there is no central inversion symmetry along the c-axis of the crystal cell. Hence, the <0001> crystal orientation GaN, grown along the c-axis, can be divided into two types according the polarization directions, i.e., Ga-polar face ([0001] direction) and N-polar face ([000−1] direction) GaN [11,16]. Since both Ga- and N-faced GaN materials have been demonstrated to have huge potential in the above application fields, it is of great significance to study the nanotribological properties of the two different faces. However, the current research topic comparing the Ga-face and N-face of GaN is mainly focused on their chemical etch properties [21,22] and the chemico-mechanical polishing (CMP) characteristics [11,15,16], meanwhile the nanotribological properties on Ga- and N-faced bulk GaN surfaces remain unclear.

In this work, the nanotribological properties of Ga- and N-faced GaN surfaces were, respectively, studied through nanoscratch experiments. Firstly, the hardness and elastic moduli of Ga- and N-faced GaN samples were measured by nanoindentation tests. Secondly, the scratch-induced material deformation and frictional properties of the two faces of GaN dominated by mechanical interaction were investigated by using nanoscratch experiments under different loading conditions. To further investigate the mechanism of difference in nanotribological properties between Ga- and N-faced GaN samples, high resolution transmission electron microscopy (HRTEM) observation and X-ray photoelectron spectroscopy (XPS) analysis were performed. This work may help us to understand the nanotribological properties of the Ga-polar and N-polar-faced bulk monocrystalline GaN, fabricated by the conventional techniques.

## 2. Materials and Methods 

### 2.1. GaN Material

For the current application of devices, monocrystalline GaN material is generally prepared on a heterogeneous substrate (e.g., sapphire, silicon carbide, and silicon) through MOCVD method [23]. Due to the lattice mismatch and thermal expansion coefficient mismatch, GaN grown epitaxially on these heterogeneous substrates generally have a high defect density (10^8^–10^10^ cm^−2^ [23]), which would, to some degree, reduce the device performance and cause the poor heat dissipation. In our study, the wurtzite GaN samples were prepared by applying the hydride vapor phase epitaxy (HVPE) technique to the GaN homogeneous substrate, and thus, such homoepitaxial bulk GaN samples would exhibit a lower dislocation density and superior performance [15]. These as-received n-type undoped c-plane GaN wafers with a thickness of 0.36 mm were purchased from Hefei Crystal Technical Material Co., Ltd (Hefei, China). The defect density of these GaN samples was measured to be below 5 × 10^6^ cm^−2^ by cathode fluorescence (CL) spectrum. Before the experiments, the GaN samples were ultrasonically cleaned in acetone, alcohol, and deionized water successively to remove the surface impurity.

**Figure 1:**
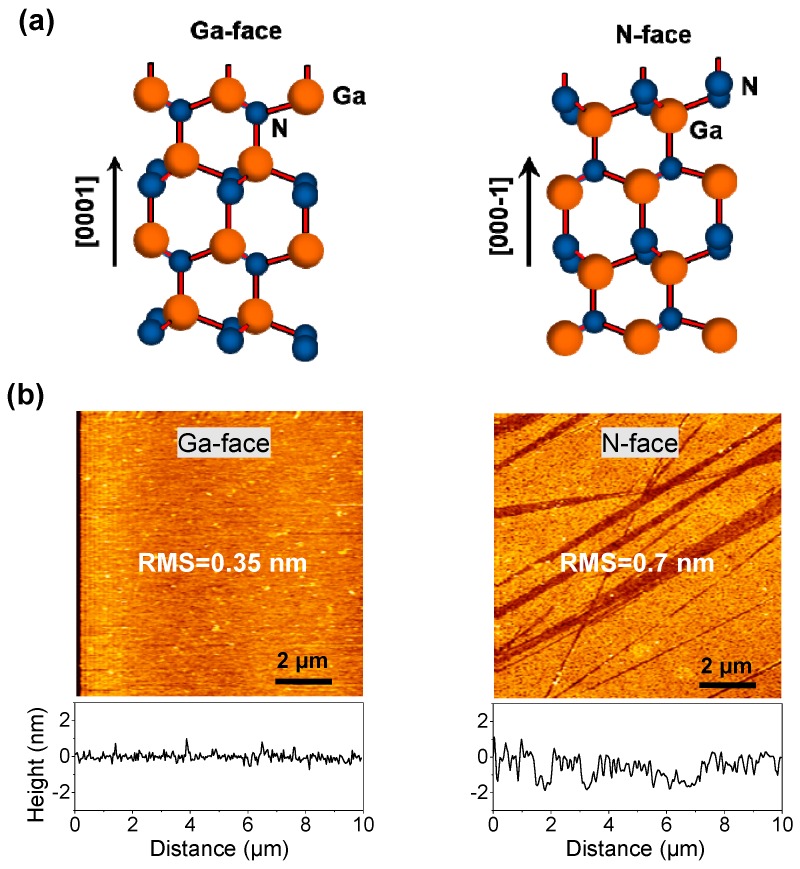
(**a**) Schematic diagram showing the different atomic arrangements of Ga- and N-faced c-plane gallium nitride (GaN) with the wurtzite crystal structure; (**b**) atomic force microscope (AFM) surface images and cross-sectional profiles of the Ga-face and N-face of a GaN sample in our experiments.

These c-plane GaN samples were double side polished by the conventional CMP technique. According to the polarization direction, the two sides of the GaN sample were separately (0001) Ga-face and (000−1) N-face, i.e., customarily called Ga-faced GaN and N-faced GaN, respectively. The different atomic arrangements of Ga-faced GaN and N-faced GaN are shown in Figure 1a. By using an atomic force microscope (AFM, AFM5300E, Hitachi, Tokyo, Japan), the surface topography and root mean square roughness (RMS) of the GaN samples were characterized. Figure 1b shows the AFM images and cross-sectional profiles of the Ga-face and N-face of a GaN sample in our experiments. It was observed that there were plenty of irregular scratches on N-face of the GaN sample but no visible scratches on Ga-face; the corresponding RMS values over 10 μm × 10 μm area were measured to be 0.35 nm for the Ga-face and 0.7 nm for the N-face, respectively. Normally, polishing on N-face of GaN has been found to be more effective than that on Ga-face of GaN [11,16], while it is often more difficult to control the surface roughness of N-face during the CMP or wet etching process [24,25]. 

### 2.2. Nanoindentation and Nanoscratch Experiments

Nanoindentation and nanoscratch experiments were carried out on both Ga- and N-faced GaN samples by a nanomechanics measurement system (TI950, Hysitron Inc., Eden Prairie, MN, USA). The nanoindentation tests were performed by a diamond Berkovich nanoindenter with a curve radius of ~250 nm. The elastic modulus and hardness as a function of the penetration depth were obtained through the combination of continuous stiffness measurements (CSM) method [26] and the widely accepted theory proposed by Oliver and Pharr [27]. Nanotribological properties of Ga- and N-faced GaN samples were studied by nanoscratch experiments with a spherical diamond nanoindenter (nominal radius *R*≈1 μm). The scratching distance was 10 μm and the scratching velocity was ~0.667 μm/s. In order to preferably compare the scratching properties of Ga-face and N-face, we performed the nanoscratch experiments along the [10−10] direction on all samples, which could exclude the possible influence of crystal orientation on the scratching properties. All of the AFM images were scanned in contact mode by silicon nitride probes (MLCT, Bruker Corp., Billerica, MA, USA) with a spring constant of ~0.1 N/m and nominal radii of around 20 nm 

### 2.3. HRTEM and XPS Characterization

Microstructural characteristics of the surface and subsurface layers of Ga- and N-faced GaN samples were detected by cross-sectional transmission electron microscopy (XTEM, Jeol JEM-2800, Tokyo, Japan) analysis. The XTEM lamellae of the observation areas were cut along the (10−10) crystal plane by using a focused ion beam system (FIB, Helios NanoLab 660, FEI, Hillsboro, OR, USA). Before FIB cutting, an epoxy polymer protective layer with a thickness of at least 100 nm was deposited onto the GaN sample surface to protect the surface from the possible structural damage results of the subsequent attack of the high-energy ion beam. The HRTEM lattice images were obtained from the surface and subsurface of the GaN samples within 10 nm × 10 nm areas. XPS analysis was performed on the surfaces of Ga- and N-faced GaN samples employing an XPS system (Thermo Scientific K-Alpha+, East Grinstead, UK) with an Al Kα x-ray excitation source (1486.6 eV).

## 3. Results and Discussion

### 3.1. Nanoindentation Tests on Ga- and N-Faced GaN Samples 

**Figure 2:**
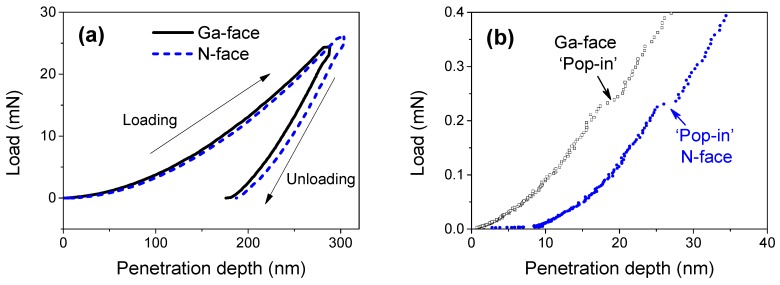
Nanoindentation tests on Ga- and N-faced GaN samples. (**a**) Loading and unloading curves of nanoindentation; (**b**) ‘Pop-in’ phenomena during the initial loading stage.

For the purpose of studying the nanotribological properties of Ga- and N-faced GaN surfaces rubbed against a spherical diamond nanoindenter, the basic mechanical properties (elastic modulus and hardness) first needed to be characterized through nanoindentation tests. Figure 2a shows the standard nanoindentation curves obtained from Ga- and N-faced GaN samples with the maximum penetration depths *h*_max_ of ~300 nm, indicating a difference between Ga- and N-faced GaN samples. To further investigate the transition of elastic deformation to elastoplastic deformation for the two different faces, the initial loading curve with penetration depth below 40 nm is plotted in Figure 2b. It was found that the obvious ‘pop-in’ phenomena [28] occurred on both the two faces, and the critical normal load to trigger the ‘pop-in’ event was ~200 μN. Generally, the ‘pop-in’ phenomena can signify the initiation of the plastic deformation, where the contact pressure exceeds a critical value and dislocation is produced inside the monocrystalline GaN. We also noticed that when the ‘pop-in’ event was taking place, the penetration depth for N-faced GaN was deeper than that for Ga-faced GaN.

**Figure 3:**
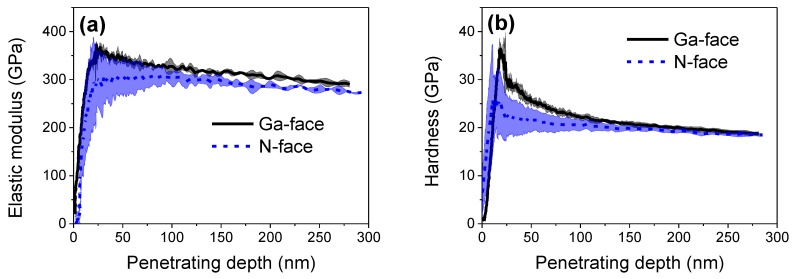
Elastic modulus (**a**) and hardness (**b**) vs. indentation depth on Ga- and N-faced GaN samples. The filled areas under the curves show the standard deviation for multiple repeated experiments.

Figure 3 shows that the comparison of elastic modulus and hardness varied with the penetration depth for Ga- and N-faced GaN samples. It indicated that (i) when the penetration depth was below 50 nm, both the elastic modulus and hardness significantly changed with the penetration depth, and then gradually became stable; (ii) the Ga-faced GaN sample exhibited a higher elastic modulus and hardness than the N-faced GaN sample. The further calculation result indicated that the stable elastic modulus for Ga-faced GaN sample (*E*_Ga-face_ = 317 ± 16 GPa) was higher than that of the N-faced sample (*E*_N-face_ = 295 ± 10 GPa), while the stable hardness for the Ga-faced GaN was similar to that for the N-faced sample (*H*_Ga-face_ ≈ *H*_N-face_ ≈ 20 GPa). 

### 3.2. Nanotribological Properties on Ga- and N-Faced GaN Samples

**Figure 4:**
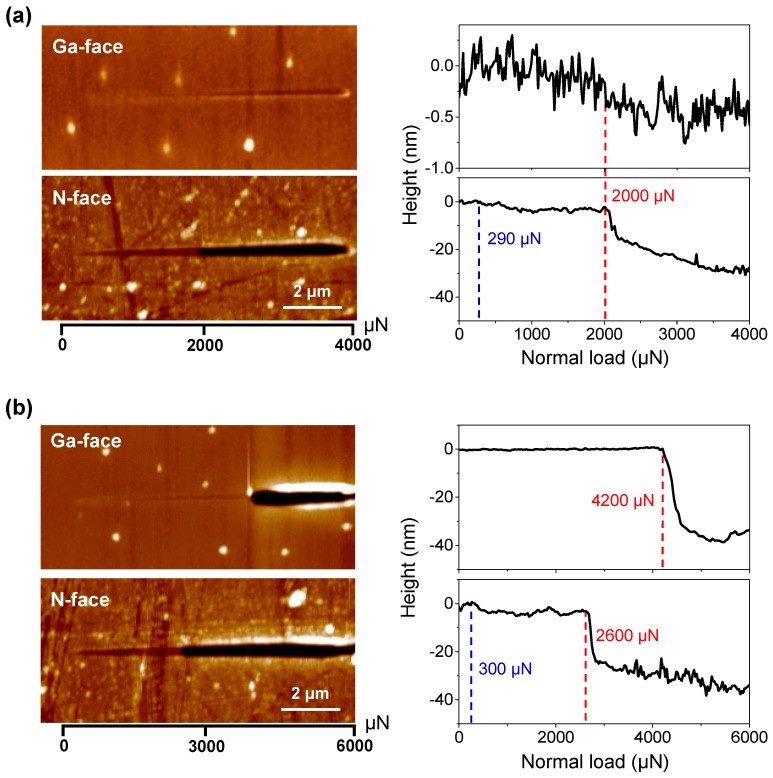
Nanoscratch tests on Ga- and N-faced GaN samples under the progressive normal load. (**a**) The progressive normal load ranged from 0 to 4000 μN. (**b**) The progressive normal load ranged from 0 to 6000 μN. The left AFM images show the morphology of the scratches and the right curves show the depth of scratch-induced groove with respect to the normal load. The scratching distance was 10 μm.

Nanoscratch experiments were separately carried out on Ga- and N-faced GaN samples under the same scratching condition. Figure 4a shows the in situ scratch profiles of Ga- and N-faced GaN samples under the progressive normal load from 0 to 4000 μN (alternatively called ramped load of 4000 μN), which suggests that the surface nanoscratch properties between the two faces of GaN are much different. For N-faced GaN sample, with the normal load increased from 0 to 4000 μN, the depth of scratch-induced groove gradually increased from 0 to 30 nm and the material deformation behavior can be divided into three distinct stages; that is, elastic deformation stage, elastoplastic deformation stage, and material removal (wear) stage. Figure 4a shows that the critical normal load for the transition changed from pure elastic to elastoplastic is ~290 μN, where the corresponding maximum Hertzian contact pressure is estimated as ~15 GPa, approaching the reported yield strength of 15 GPa for GaN [29]. When the normal load exceeded ~290 μN, a groove formed with the inconspicuous ‘pile-up’ phenomena due to the plastic deformation of GaN material. When the normal load increased to ~2000 μN, obvious material removal with a lot of wear debris on both sides of the scratch could be observed, resulting in a sharp increase of the groove depth from ~5 nm to ~16 nm. At this moment, the scratching behavior of N-faced GaN exhibited a change from ductile-brittle transition region (dominated by elastoplastic deformation) to a completed brittle region (material removal occurred), which is similar to the reported research results for the nanoscratch properties of c-axis GaN epilayers surface [20]. We noticed that Ga-faced GaN sample showed a stronger wear resistance than N-faced GaN sample: With the normal load varied from 0 to 4000 μN, no significant material removal and no such obvious three stages (the case of N-faced GaN sample) took place on Ga-faced GaN sample. Instead, only discernible plastic deformation along with groove depth lower than 1 nm could be found on the Ga-face sample surface when the normal load ranged from 2000 to 4000 μN.

To further investigate the differences of material removal between Ga- and N-faced GaN samples, another set of nanoscratch tests was conducted by increasing the maximum normal load to 6000 μN, as shown in Figure 4b. In this experiment, we found that when the normal load was further increased to ~4200 μN, obvious material removal eventually occurred on the Ga-face GaN surface and the groove depth sharply increased to ~40 nm. It should be noted that when the normal load exceeded ~4200 μN, the groove depth of the Ga-faced GaN sample was like that of the N-faced GaN sample under the same normal load. Figure 4b shows that the critical normal load to cause material removal of N-face is ~2600 μN when scratching under the progressive load varied from 0 to 6000 μN, while the critical normal load to cause material removal is only 2000 μN when scratching under the progressive load varied from 0 to 4000 μN (Figure 3b). Given that the scratching distance was 10 nm and the scratching velocity was 0.667 μm/s in our experiments, the loading rate for nanoscratch under the progressive load of 0–4000 and that under the progressive load of 0–6000 μN were, respectively, 267 μN/s and 400 μN/s, which may result in the different responses of material deformation, and hence further influence the mechanical properties, and even the material removal behavior [30,31].

**Figure 5:**
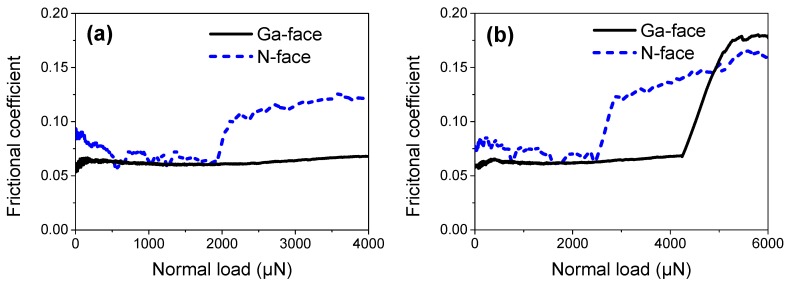
Frictional coefficient varied with the normal load on Ga- and N-faced GaN samples. (**a**) The progressive normal load from 0 to 4000 μN. (**b**) The progressive normal load from 0 to 6000 μN.

Meanwhile, the corresponding frictional coefficient varied with normal load is plotted in Figure 5. As can be seen, the GaN surface exhibited a strong wear resistance with very a low frictional coefficient when rubbed by a diamond microsphere. With the increase in normal load, the variation trend of the frictional coefficient on Ga- and N-faced samples was like the variation trend of the nanoscratch depth. It is interesting to note that during the elastic and elastoplastic deformation stages, the frictional coefficient of the Ga-faced GaN surface (with the average value of ~0.06) was lower than that of the N-faced GaN surface (with the average value of ~0.075). When the nanoscratch-induced material removals occurred, the frictional coefficient suddenly increased to two times that of the frictional coefficient before wear, both on Ga- and N-faced GaN surfaces. Moreover, the frictional coefficient would continue to increase when increasing the normal load. When the normal load was above 4200 μN, the frictional coefficient of the Ga-faced GaN surface was higher than that of the N-faced GaN surface (shown in Figure 5b). It can be concluded that the frictional coefficient of the GaN surface is tightly connected with the nanoscratch-induced material deformation of GaN. Once the material removal takes place, the frictional coefficient would rapidly increase, and the more severe the material removal, the greater the friction coefficient.

**Figure 6:**
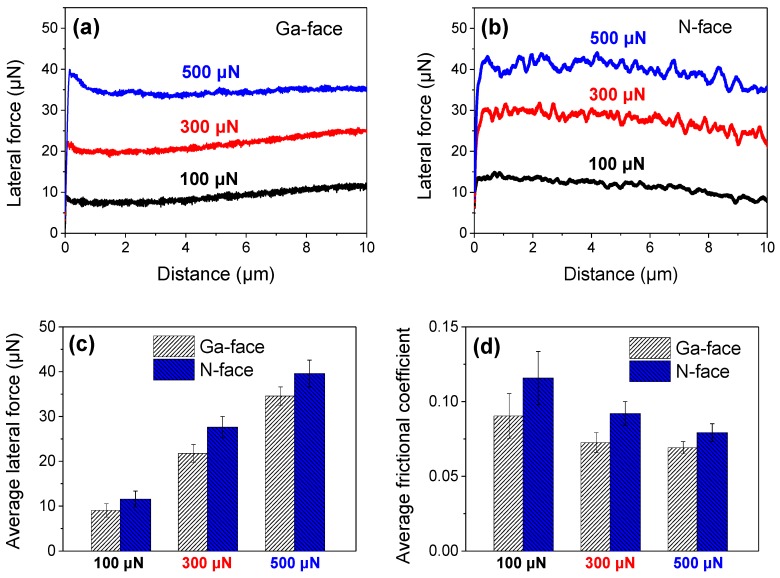
Comparison of the lateral force on Ga- and N-faced GaN samples under different constant normal loads of 100 μN, 300 μN, and 500 μN, respectively. (**a**) Lateral force vs. scratching distance of the Ga-faced sample. (**b**) Lateral force vs. scratching distance of the N-faced sample. (**c**) Comparison of the average lateral forces of the Ga- and N-faced GaN samples under different constant normal loads. (**d**) Comparison of the average frictional coefficients of the Ga- and N-faced GaN samples under different constant normal loads.

Figure 6 shows the comparison of the lateral force (frictional force) on Ga- and N-faced samples during a 10 μm distance nanoscratch process, under a series of constant normal loads of 100 μN, 300 μN, and 500 μN, respectively. With the normal load increased from 100 to 500 μN, the maximum Hertzian contact pressure ranged from 11 to 18 GPa, under which the nanoscratch properties were dominated by the elastoplastic deformation, and material removal almost cannot occur on the monocrystalline GaN surface. It could be also noticed that the lateral force versus scratching distance curve of the Ga-faced GaN sample stayed relatively stable (Figure 6a), but the curve of the N-faced GaN sample fluctuated obviously with respect to the sliding time (Figure 6b), which was mainly ascribed to the different surface roughness of Ga- and N-faced GaN samples (shown in Figure 1b). To preferably suppress the influence of static portion, Figure 6c,d, respectively, show the statistical average values of lateral force and frictional coefficient under different normal loads obtained over the course of the scratching process with a distance of 10 μm, which clearly indicated that the lateral force and frictional coefficient of the Ga-faced GaN sample were lower than those of the N-faced GaN sample when performing nanoscratch tests under the same normal load. Figure 6d also indicated that the frictional coefficient decreased with increasing the normal load, both on the Ga-face and the N-face. In addition, we observed the frictional coefficient when scratching under the constant normal load was higher than that under the progressive normal load, both for Ga-faced (0.07–0.09) and N-faced (0.08–0.1) GaN samples. Such a difference was probably caused by two different loading methods. Overall, it suggested that at the pure elastic and elastoplastic deformation stages of material, the Ga-faced GaN sample exhibited higher frictional force and frictional coefficient compared to the N-face GaN sample when rubbed by a diamond indenter under the same experimental condition.

### 3.3. Explanation of the Difference in Nanotribological Properties for Ga- and N-Faced GaN Samples

**Figure 7:**
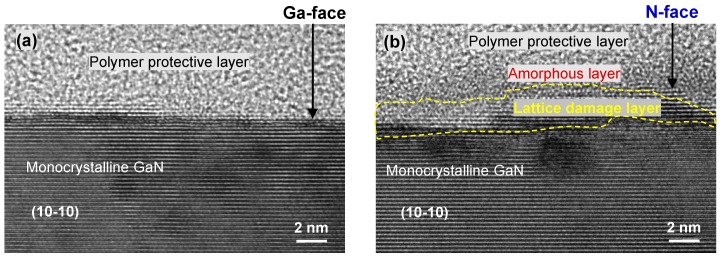
HRTEM images of the cross-section of Ga-faced GaN sample surface (**a**) and N-faced GaN sample surface (**b**). The cross-section was the (10−10) crystal plane of hexagonal wurtzite GaN.

Due to the fact that diamond is highly chemically inert, the frictional force and material removal behavior of the GaN samples in our experiments were governed by the mechanical interaction between GaN and the diamond indenter. Given that Ga- and N-faced GaN samples did not show significantly different mechanical properties (elastic modulus and harness, shown in Figure 3), we thought the obvious difference in nanotribological properties between the two faces of GaN was not only related to their mechanical properties (dominated by the different atomic arrangements of Ga- and N-faced GaN), but also attributed to some factors else. To further investigate the differences of nanotribological properties on Ga- and N-faced GaN samples, HRTEM observation was performed on the cross-section ((10−10) crystal plane) of the surface and subsurface in GaN samples. As shown in Figure 7, an inconspicuous amorphous layer with a thickness of 1–2 nm (probably a native oxide layer, but difficult to be clearly recognized by TEM) and a slight lattice damage layer with a thickness of ~2 nm were observed on the N-faced GaN sample surface, while such inconspicuous structural layers could not be observed on the Ga-faced GaN sample surface at all. 

**Figure 8:**
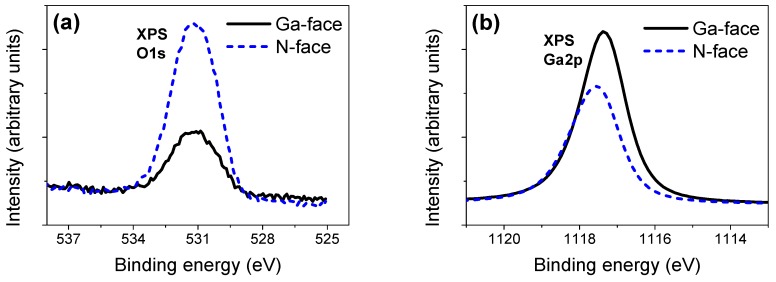
XPS analysis of the surfaces of Ga- and N-faced GaN samples. (**a**) Comparison of O1*s* XPS spectra (core level of ~532 eV) on Ga-face and N-face. (**b**) Comparison of Ga2p XPS spectra (core level of ~1117 eV) on Ga-face and N-face.

It has been known that N-face is more chemically active than the chemically inert Ga-face due to the nonreverse crystallographic symmetry of c-plane GaN [11,16]. For example, on the Ga-face, each nitrogen atom existing on the surface after removing the top Ga-layer has three dangling bonds; by sharp contrast, each nitrogen atom in the top layer has only one dangling bond on the N-face [21]. Employing density functional theory, Tosja et al. found that the N-face surface generally exhibits a higher activity towards oxygen adsorption compared to the Ga-face surface in terms of the different surface atomic structures [32]. Combined with XPS analysis of the oxygen-related peak structures, Eickhoff et al. [33] concluded that the N-face of GaN is more easily naturally oxidized to form a native oxide layer (Ga*_x_*O*_y_*-like structure) on its surface after storage in the air, which is in good agreement with previous theoretical predictions [32]. Moreover, we also conducted the XPS analysis for our Ga- and N-faced samples. As seen in Figure 8a, N-face showed a more intensely O1s XPS peak structure than N-face, indicating N-face had a higher amount of oxygen. Figure 8b shows a peak shift of 0.3 eV from Ga-N to Ga-O in the Ga2p peak on the N-face relative to the Ga-face (Figure 8b), which could, to some extent, indicate the more obvious formation of the Ga*_x_*O*_y_*-like layer on N-face surface. In addition, the calculation result indicated that the oxygen content of the N-face surface (15.6 at.%) was higher than that of Ga-face surface (7.7 at.%), while the nitrogen content of N-face (40.4 at.%) was decreased compared to the Ga-face surface (48.1 at.%), suggesting that part of the Ga-N bonding was highly likely to be replaced by Ga-O bonding on N-face surface. Our XPS analytical result supports the viewpoint of Ref. 32 and Ref. 33 [32,33], i.e., N-face of GaN indeed have a higher activity towards oxygen and a Ga_x_O_y_-like layer could predominately form on the N-face surface. 

Hence, N-face of GaN is more easily naturally oxidized to form a native oxide layer on its surface when exposed in the atmospheric environment or during the CMP process. Due to its much lower hardness, the native oxide layer on N-faced GaN surface is supposed to have a weaker wear resistance in comparison to monocrystalline GaN. On the other hand, since the N-faced GaN sample has a higher material removal rate than that of the Ga-faced sample, there tends to be a worse surface quality on N-face than that on Ga-face during the CMP process [11,16,24] (also supported by the measured RMS values in Figure 1b). Meanwhile, the stronger mechanochemical, involving more material removal may, favor the generation of lattice damage. Therefore, we speculated that the observed slight lattice damage of the N-faced GaN sample was probably produced in the CMP process.

To sum up, we thought that native oxide layer, as well as the lattice damage layer of the N-faced GaN sample surface, had weaker mechanical properties and wear resistance than the monocrystalline GaN without lattice damage, and thus, when the diamond indenter penetrated the GaN sample to a small depth (wear just occurred in the surface), it would lead to the difference in nanotribological properties between the two faces of GaN. Once the surface oxide layer and the damage layer were worn through and the indenter penetrated the subsurface, the nanotribological properties of Ga- and N-faced GaN samples were mainly ascribed to the intrinsic atomic arrangements of Ga- and N-faced GaN. This work also suggests that the lattice damage could be easily generated on N-faced GaN surface through the conventional CMP techniques. Such damage can, to some extent, reduce the wear resistance of GaN substrates, influencing the device performance and service life. Hence, the CMP techniques for the N-faced GaN substrate free of lattice damage should be further exploited.

## 4. Conclusions

In this study, nanotribological properties of Ga- and N-faced GaN samples were, respectively, studied by nanoscratch experiments. Nanoindentation results revealed that the elastic modulus of the Ga-faced GaN sample was a little higher than that of the N-faced GaN sample. With the increase of normal load, the nanoscratch-induced deformation of GaN went through the three stages of pure elastic, elastoplastic, and material removal, on both the Ga-faced and N-faced GaN surfaces. This indicated that the Ga-face exhibited a stronger wear resistance compared to N-face, and the critical normal load required to cause material removal of the Ga-faced GaN was almost two times higher than that of N-faced GaN. Both the Ga-face and N-face showed a rather low frictional coefficient at elastic and elastoplastic stages. Combined with HRTEM observation and XPS analysis, we speculated that the differences in nanotribological properties between Ga- and N-faced samples in our experiments may be related not only the intrinsic atomic arrangement due to the nonreverse crystallographic symmetry of c-plane wurtzite GaN, but also to a 3–4 nm thick structure composed of a lattice damage layer and a native oxidation layer on N-faced GaN surface. The slight lattice damage layer (probably generated during the CMP process), as well as the native oxide layer on N-face could reduce the wear resistance of the GaN sample. Once these layers were worn through, the differences in nanotribological properties between Ga- and N-faced GaN samples were mainly dominated by the different atomic arrangements, owing to the nonreverse crystallographic symmetry of c-plane GaN.
